# Supplementary material for: Patterns of Intron Gain and Loss in Fungi
Source: PLoS Biol. 2004 Nov 30;2(12):e422. doi: 10.1371/journal.pbio.0020422 (PMC532390; doi:10.1371/journal.pbio.0020422)
Supplement: Table S1 — Also available at http://genes.mit.edu/NielsenEtAl/. (4.3 MB ZIP). [file pbio.0020422.st001.zip › NielsenEtAl/html/1383.html]

AN6307.1.NCU06242.1.MG06367.1.FG07250.1


```
 CLUSTAL W (1.82) Multiple Sequence Alignments - Introns Inserted


Sequence 1: NCU06242.1	338 aa
Sequence 2: MG06367.1	322 aa
Sequence 3: FG07250.1	325 aa
Sequence 4: AN6307.1	360 aa
Alignment Length: 388 aa
Number Identitical Residues: 165 aa
Alignment Score (without introns) 7813


MG06367.1 	-MWFTSCLSALCLAATGALAAI-SVDDSGEVKSIG0---------------------LRT
NCU06242.1	MMLLTSALSALWLATA-AHGAV-QQDAEGNVKSIG~VSSGSSASRHSAHGTNAFFQQLRT
FG07250.1 	-MRLSSLSAALLGAFAWTTSAS-EFDDE-NIRSIS0---------------------LRT
AN6307.1  	-MLLPRFSTLLCLAGLTAVPAVNAYDGDENVKSIP0---------------------LRT
          	 * :.   : *  *   :  * .  * . :::**                       ***

MG06367.1 	HSLMQ0PYLDSDMQSRWYDFGGDTIIRTDQ2YIRLTSDRPSQMGSLWSRVPLTATNWE~I
NCU06242.1	HSLNQ0PYLDSDTQSRWYDFGGDTIIRTDQ2YIRLTSDRPSQMGNLWSKVPLTATNWE~I
FG07250.1 	HSLTQ0PYLDSDMQSRWYDFGGDTIIRTDS2YIRLTSDRPSQSGWMYSRVPLTATNWQ~V
AN6307.1  	HSLSA0PYLDSDFQSRWFDFGGDTVIRADK2YIRLTSDRPSQQGWIFSRVPLTATNWE0I
          	***   ****** ****:******:**:*. *********** * ::*:********: :

MG06367.1 	EVEFKIHGKHQLYGDGFAMWVTKGRNDVGPVFGNADKFEGLGLFFDTYKNNRPGVVFPYV
NCU06242.1	EVEFKISGKNQLYGDGFAMWLTKERGREGPVFGNQDKFEGLGIFFDTYKNNRPGVVFPYV
FG07250.1 	EVEFKISGKNQLYGDGFAMWITRQRAQQGTVFGGPDNFEGLGVFIDTYKNNRPGVVFPYV
AN6307.1  	EFEFQIHGEGNLHGDGFAMWLTKQRATQGPVFGSTDNFEGLGIFFDTYKNNRPGTSFPYV
          	*.**:* *: :*:*******:*: *   *.***. *:*****:*:*********. ****

MG06367.1 	MAMVGDGHTAYDKDTDGKGNEFAGCS~ARGIRHASIPTKFRLTYFQDKSLKLELHYKSDG
NCU06242.1	MAMVGDGRTSYDKDTDGKNQELAGCS~ARGIRHANVPTKFKLSHFQDKYLKLELQYKNEG
FG07250.1 	MAMYGDGKTSYDKSNDGKHTELAGCS~ARGIRHASIPTKLRLTYFQDKQLKLELQYKVED
AN6307.1  	MAMMGDGKTSYDQAHDGKANELAGCS0ARGLRTTSVPTKARLTYFQDKSLTLDLQYKSEG
          	*** ***:*:**:  ***  *:**** ***:* :.:*** :*::**** *.*:*:** :.

MG06367.1 	EWTTCFETDEPPT---IPQVAYLGFTAETGELSDNHDIISISAKNLYTAQPSGSKDNKKA
NCU06242.1	EWLLCFETNQPPT---IPPVAYLGFTAETGELSDNHDIISINARNLYTTAPP-----TKS
FG07250.1 	EWQTCFDLEDPPA---VPNIAYVGFTAETGELSDNHDIISVAAKNLYTQPGTSSNTGSKS
AN6307.1  	TWTNCFTLTSPETNIAIPSVAYLGFSAETGELSDNHDIISVKAQNLYNVAGS------KG
          	 *  **   .* :. ::* :**:**:**************: *:***.   .      *.

MG06367.1 	DKTKGKK----AKKVKEHKEGGSWTWMMIKIFLFIVVIGGAY-------VGYTAWKTNK-
NCU06242.1	TPGSGK-----GKKVTDQQAEGSWTWFLVKTLFFFFAVGGAY-------VGYTAYRTKQQ
FG07250.1 	SSSKGKSRKGSGKTTGGQKEGGSWTWFFTKIILFIIVAGGAY-------VGYTAYRSKA-
AN6307.1  	TGGSGPPRSADRPYRKPKKQSGSWSWFLFKVFFFFAAVRGGYDWMDRLPVQDKVFSVLDE
          	   .*   ...      ::  ***:*:: * ::*: .  *.*.  .  .*  ..:    .

MG06367.1 	RSHRF----------------------------
NCU06242.1	RSHRF----------------------------
FG07250.1 	KSHRF----------------------------
AN6307.1  	RVVDFCGLMRSGPMSESEMVKIKTLSVILYLLA
          	:   *..   :.. :.:.  . .: :      :
```
